# Haul‐Out Site Use and Connectivity of Harbour Seals Between Management Units in Southern Scandinavia

**DOI:** 10.1002/ece3.72718

**Published:** 2026-01-13

**Authors:** Javed Riaz, Kjell T. Nilssen, Martin Biuw, Even Moland, Michael Poltermann, Martin Kristiansen, Carla Freitas

**Affiliations:** ^1^ Institute of Marine Research His Norway; ^2^ Department of Climate Change, Energy, the Environment, and Water, Australian Antarctic Division Kingston Tasmania Australia; ^3^ Institute of Marine Research Tromsø Norway; ^4^ Centre for Coastal Research University of Agder Kristiansand Norway; ^5^ MARE Marine and Environmental Sciences Center Funchal Madeira Portugal

**Keywords:** biologging, haul‐out, satellite telemetry, Skagerrak, transboundary

## Abstract

Harbour seals ( *Phoca vitulina* ) have a broad distribution in coastal ecosystems across the northern hemisphere. In southern Scandinavia, a lack of spatially‐resolved data on harbour seal populations poses a major challenge for developing ecologically informed management frameworks, particularly in Norway, where populations are regulated using county‐level administrative boundaries. In this study, we use haul‐out data from 26 harbour seals tagged with GPS phone tags during the post‐moult period to provide the first assessment of connectivity and movement across management boundaries in the Skagerrak–Kattegat region of southern Scandinavia. Specifically, we examined the frequency and timing of haul‐out events relative to management units and quantified spatial networks of connectivity across national and sub‐national jurisdictions. We reveal a high degree of spatial connectivity in the region, with haul‐outs occurring over a broad, integrated network along the Skagerrak–Kattegat coastline. Generally, harbour seals in the region had a high probability of performing cross‐boundary haul‐out events, with individuals repeatedly transitioning across distinct Norwegian management units, as well as transnationally between Norway, Sweden and Denmark. This study offers critical insights into harbour seal movement ecology in this data‐limited region, whilst also addressing an important topic of applied management. We demonstrate that the current management units in the Norwegian Skagerrak may not adequately reflect the spatiotemporal scales of harbour seal movement. Importantly, these findings can complement forthcoming genetic data and support efforts to redefine management units in the area. More broadly, our study illustrates how telemetry‐based assessments of spatial connectivity can provide a powerful tool to inform management frameworks for other wide‐ranging marine species facing similar conservation challenges.

## Graphical abstract

Effective management of wild populations requires knowledge of their movement patterns and connectivity across administrative borders. Using telemetry data from harbour seals in southern Scandinavia, we show that individuals routinely move across national and regional jurisdictions, creating an integrated network of haul‐out sites. These findings highlight the importance of incorporating spatial connectivity into management planning and provide valuable information that can help inform and evaluate the redesign of management units in the region.

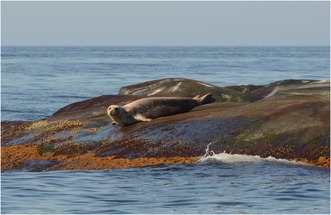

## 1 Introduction

Understanding the spatial ecology of marine species is vital for effective marine conservation and management (Lennox et al. 2019). Insights into the movement behaviour, habitat use and connectivity of wild populations can assist policy makers and environmental managers to make informed and evidence‐based decisions (Heylen and Nachtsheim 2018; Dunn et al. 2019; Sequeira et al. 2019). There are numerous examples from around the world demonstrating how movement data collected by animal‐borne data loggers can be used to guide area‐based management approaches, including the spatiotemporal design of marine protected areas and the structuring of management units to regulate anthropogenic activities (e.g., commercial fisheries and population controls) (Hays et al. 2019; Carter et al. 2022).

For area‐based conservation and management frameworks to be effective, they must operate at ecologically relevant scales, accounting for the spatial dynamics of target populations (Choi et al. 2019; Allen and Singh 2016; Conners et al. 2022). However, this can be particularly challenging to achieve for large and mobile marine predators, such as seabirds and marine mammals, which commonly travel large distances and traverse geospatial and political boundaries (Runge et al. 2014; Reisinger et al. 2022; Harrison et al. 2018). Spatial planning and management of these marine taxa are inherently more complex, with outcomes contingent on the cooperation and coordination between national and/or sub‐national management agencies (Kark et al. 2015; Harrison et al. 2018).

Harbour seals ( *Phoca vitulina* ) are the most widely distributed pinniped species in the northern hemisphere, ranging across temperate and arctic coastal habitats (Blanchet et al. 2021; Teilmann et al. 2023). In continental Europe, they have a ubiquitous distribution between France and northern Norway (approximately 48°–70° N), where they use coastal habitats to moult, breed and haul out. Harbour seals are typically characterised by their localised distribution and fine‐scale population structuring, seldom moving beyond 50 km from natal haul‐out/breeding sites (Dietz et al. 2013; Vincent et al. 2017; Sharples et al. 2012; Olsen et al. 2014; Huon et al. 2021). However, several studies have also found evidence of more wide‐ranging dispersal (> 100 km) and regional‐scale connectivity (Sharples et al. 2012; Carter et al. 2022; Olsen et al. 2017; Jones et al. 2015). Across the various European jurisdictions, harbour seal populations are managed using different area‐based approaches, ranging from full protection to controlled hunting (Blanchet et al. 2021; Teilmann et al. 2023). Spatially resolved ecological data are therefore critically important to support the assessment, monitoring and management of European harbour seal populations (Jones et al. 2015; Olsen et al. 2014, 2017).

The Skagerrak–Kattegat marine ecosystem in southern Scandinavia is a region where there are numerous harbour seal breeding/haul‐out sites. The region consists of three national jurisdictions, Norway, Sweden and Denmark—which adopt different area‐based harbour seal management regimes. In Sweden and Denmark, harbour seals are protected under the EU Habitat Directive and national legislation, although preventative and licensed hunting can be granted to reduce damage to fishery catches and fishing gear (Teilmann et al. 2023; Carroll et al. 2025; ICES 2023). However, in Norway, harbour seal populations are regulated through harvest quotas to maintain politically agreed target levels (ICES 2023). Population status relative to these target levels is established through population surveys conducted every five/six years. Regulation occurs at a sub‐national scale, with hunting quotas issued to individual counties according to count estimates of hauled out individuals during the moulting period. This means that harbour seal management units in Norway are delineated by administrative boundaries rather than any biological or ecological justification (Blanchet et al. 2021; Nilssen et al. 2010; NAMMCO 2025).

There is increasing recognition of the need to evaluate the efficacy of these administrative management units in Norway, and potentially revise the management framework to be more consistent with population‐level distribution and dynamics (NAMMCO 2025; Blanchet et al. 2021). However, these objectives are hindered by a paucity of spatially resolved ecological data on harbour seal populations, notably in the Norwegian Skagerrak. Genetic analyses of harbour seals hauled out along the Swedish and Danish coastlines have identified distinct populations in the region, which has helped to establish several sub‐national units for conservation, monitoring and management purposes (i.e., Skagerrak, Kattegat, Limfjord, Wadden Sea, Western Baltic and Baltic proper) (Olsen et al. 2014). Research efforts are currently being undertaken to extend these molecular analyses to haul‐out sites along the Norwegian coast, including the Norwegian Skagerrak—offering a potential framework for distinguishing biologically meaningful management units (M. Quintela, personal communication). These genetic tools can yield powerful insights into population structuring over longer‐term (evolutionary) timescales, providing estimates of per‐generation migration rates and coarse spatial delineations of population units (Olsen et al. 2017; Carroll et al. 2020). However, genetic investigations do not provide information on movement patterns over shorter (intra‐generational) and more contemporary timescales relevant for conservation and management—particularly when it comes to marine resource management and environmental assessment of maritime developments. For instance, harbour seal populations considered genetically distinct may still overlap in their haul‐out or foraging distribution, constituting a single ecological unit for the purposes of area‐based management (Steinmetz et al. 2023; Carroll et al. 2020; Olsen et al. 2017). Satellite telemetry data help bridge this knowledge gap, providing critical insights into harbour seal movement patterns, habitat use and intra‐generational connectivity directly relevant to population‐level conservation and management (Carroll et al. 2020; Sharples et al. 2012; Reisinger et al. 2022; Giménez et al. 2018).

In this study, we leverage satellite telemetry data of harbour seals tagged in the Norwegian Skagerrak to investigate haul‐out patterns and spatial connectivity in relation to administrative management boundaries in the region. To achieve this, we use GPS phone tags with an integrated wet/dry sensor for recording haul‐out activity. The objectives of this study were twofold:

- Evaluate the efficacy of the current management delineations in the Skagerrak through quantifying the probability, frequency and time taken for individuals to perform haul‐outs across different management areas; and
- Provide spatial information to support the development of ecologically‐relevant management boundaries by identifying networks of local‐ and regional‐scale connectivity between haul‐out sites.

Through this work, we aim to advance understanding of harbour seal haul‐out patterns in southern Scandinavia and generate information on the spatial dynamics of populations in the region, which is ultimately required to develop robust and evidence‐based management frameworks.

## 2 Materials and Methods

### 2.1 Seal Capture and Handling

**FIGURE 1:**
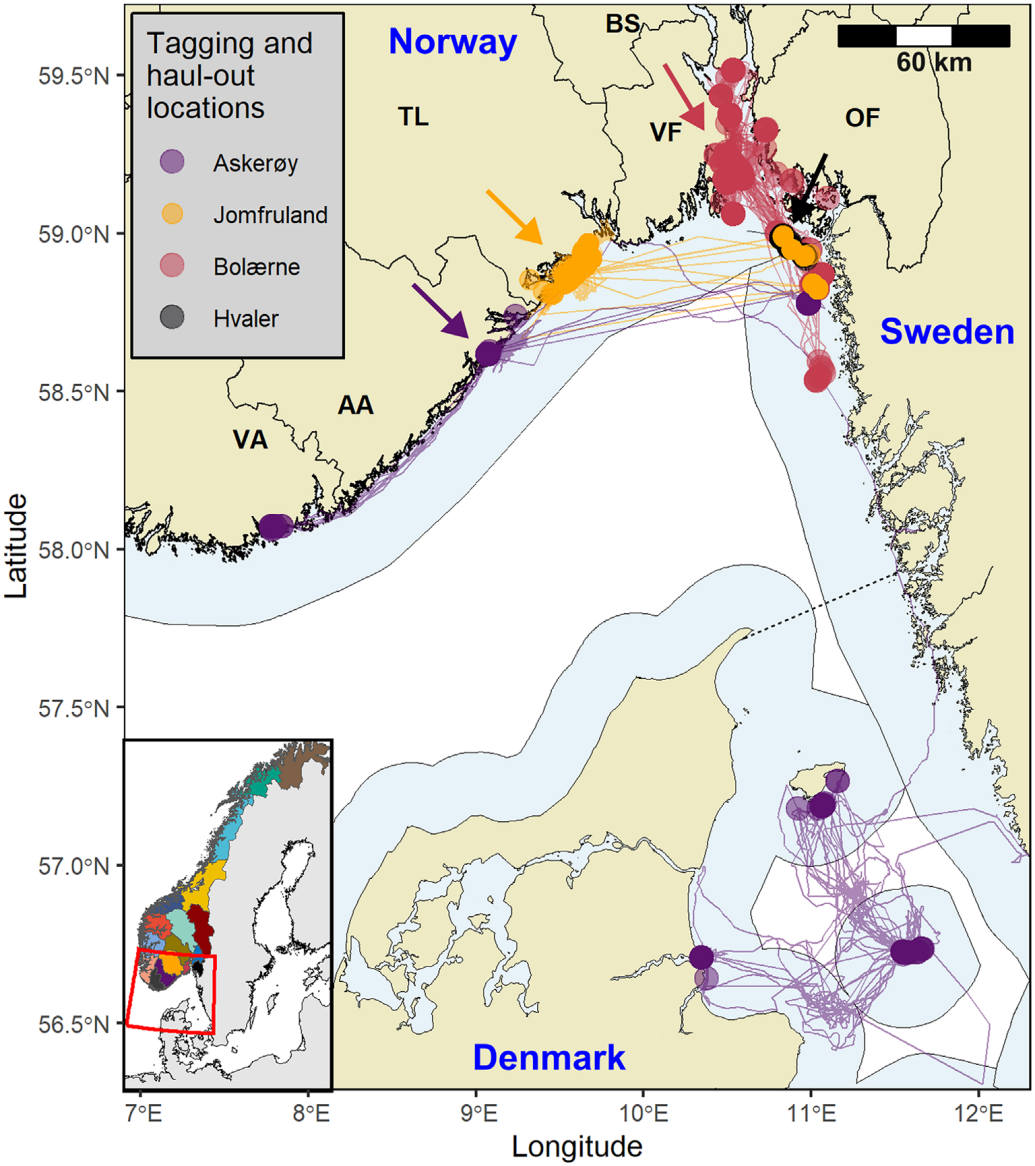
Haul‐out locations of harbour seals (*n* = 26) in the Skagerrak–Kattegat region of southern Scandinavia. Each circle represents a unique haul‐out event, coloured by the location of their initial GPS phone tag deployment. Tagging locations are indicated by coloured arrows. Corresponding coloured lines illustrate the raw at‐sea locations provided by the GPS onboard the phone tags (beyond the scope of this study but displayed for illustrative purposes). Land (beige shaded area) and maritime (blue shaded area) features on the map represent the terrestrial and territorial water boundaries of Norway, Sweden and Denmark. Norwegian land boundaries are further illustrated at the county‐level (as per 2017), consistent with Norway's harbour seal management units (VA = Vest‐Agder; AA = Aust‐Agder; TL = Telemark; BS = Buskerud; VF = Vestfold; OF = Østfold). The black dashed line represents the maritime division between the Skagerrak and Kattegat. Inset panel in the bottom left corner displays the study region (red box) and all 18 Norwegian counties (coloured shaded areas).

Harbour seal tagging efforts were conducted along the Norwegian Skagerrak coastline between the years 2017–2022. A total of 28 harbour seals were captured during the post‐moult period (August—November), with efforts distributed across four sites (Askerøy in Tvedestrand municipality, in Aust‐Agder County; Jomfruland in Kragerø municipality, in Telemark County; Bolærne in Færder National Park, in Vestfold County; and Hvaler in Ytre Hvaler National Park, in Østfold County) (Figure 1; Table S1). The tagging locations in Aust‐Agder and Telemark represent the primary known haul‐out sites for this species, based on seal counts conducted during the moulting season (Carroll et al. 2025). However, in Vestfold and Østfold, multiple haul‐out sites span over a larger area. Haul‐out sites in the region consist of small rocky islands, surrounded by seagrass or sand, with typically 10–50 individuals per island (Van Meurs et al. 2024). All tagged seals were categorised by sex (male or female) and measured (weight, standard length and girth).

To track their movements, seals were equipped with GPS phone tags (SMRU Instrumentation, University of St Andrews, UK). First generation (Gen1) GPS phone tags were used in 2017 and 2019, while tags used from 2020 were second generation (Gen2) GPS phone tags, which have no external antenna. Tags were attached dorsally to the upper shoulder region of each seal using Loctite 422 glue (Henkel Adhesive Technologies, UK). After full recovery from sedation (approximately 20 min after injection), individuals were allowed to voluntarily enter the water. The capture and handling of harbour seals in Norwegian waters was permitted by the Norwegian Food Safety Authority (permit IDs 8710, 19628 and 27565).

The GPS phone tags combine GPS‐quality locations with data transfer via the GSM mobile phone network (McConnell et al. 2004). These devices were equipped with a Fastloc GPS system that attempts to obtain a location at every surfacing event, with no duty cycle. Additionally, tags provided information on haul‐out events (the focus of this study), which were based on integrated precision pressure and wet/dry sensors. A haul‐out event was designated as any period where the wet/dry sensor was continuously dry for ≥ 10 min and ended when the sensor was wet for at least 40 s. The tags recorded the start and end times for each individual haul‐out event, as well as its location.

### 2.2 Haul‐Out Data Processing

Movement data received from the GPS phone tags were downloaded, and all subsequent data analyses were performed using R statistical software (R Core Team 2024). Individuals with less than 10 days of movement data were removed from our analysis (*n* = 2 individuals). The final dataset contained movement information for 26 individuals (Table S1). Analogous to other studies, consecutive haul‐out events that had end and start times (i.e., the inter‐haul‐out period) separated by < 10 min were merged and treated as a single haul‐out event (Hamilton et al. 2014).

Geospatial and political boundaries relevant to harbour seal management in the region were compiled. In Norway, there are 13 different sub‐national management units delineated by county‐level boundaries (refer to Figure 1). Within these counties, harbour seals may be hunted according to annual quotas set by the Norwegian Directorate of Fisheries. These quotas are based on population counts, as well as other ecological and social considerations (LOVDATA 2010). Hunting licences are issued by county municipalities and suspended once quotas are filled. Designated hunting periods run from January to April and from August to September, thereby protecting the breeding season from May to July (LOVDATA 2010). For the purpose of this study, Sweden and Denmark were considered as management units, without sub‐national delineations.

Harbour seal haul‐out data were spatially allocated to the Norwegian, Swedish and Danish management boundaries in the region, with each haul‐out event assigned to the respective administrative management unit in which it occurred. Individuals were also given unique ID labels based on the administrative management unit within which they were initially tagged (Table S1). For each individual seal, haul‐out events were assigned a binary value (0 or 1) based on whether the preceding haul‐out occurred in a different management area (1 = cross‐boundary haul‐out or 0 = no cross‐boundary haul‐out).

### 2.3 Cross‐Boundary Haul‐Out Analysis

Using the management‐relevant haul‐out delineations described above, we examined the individual‐specific and population‐level proportion of cross‐boundary events. We also examined temporal trends in haul‐out events, including the proportion of individuals that had performed a cross‐boundary haul‐out by 1 January (±1 week), which coincides with the commencement of the annual harbour seal hunting period in Norway.

To statistically examine the probability of harbour seals hauling out in different management units over time, we fit a Cox proportional hazards mixed effects model (CPH), following an approach adapted from Freitas et al. (2008) and implemented using the ‘*coxme*’ package (Therneau 2024). The model estimated the hazard of seals crossing management boundaries between haul outs, as a function of the departure management jurisdiction and body weight. Weight was included as a covariate under the assumption that an individual's haul out and movement patterns may vary with size, maturity, and/or overall fitness (Vincent et al. 2017; Hamilton et al. 2014). While sex may also influence seal behaviour, it was not included as a covariate due to collinearity with weight (males in the dataset were generally heavier than females) and insufficient representation across tagging locations (Table S1). The CPH model was configured as:

$$h\left(t\right)=h_{0}\left(t\right)\cdot \exp\left(\beta_{1}\cdot X_{1}+\beta_{2}\cdot X_{2}+b\right)$$

The hazard function $h\left(t\right)$ represents the probability of performing a cross‐boundary haut‐out event at time t. It is based on information on the seal's status (1 = changed management jurisdiction, 0 = did not change) and the corresponding date. Seal status was treated as censored at the time of each cross‐boundary haul‐out event, as well as at the end of the tracking period for individuals that never changed management jurisdiction. The term $h_{0}\left(t\right)$ represents the baseline hazard function; $X_{1}$ and $X_{2}$ are the fixed effects of the departure management jurisdiction and seal weight, respectively, with $\beta_{1}$ and $\beta_{2}$ the corresponding coefficients; and b represents the random effect of seal ID, used to account for individual variability and repeat events per individual. In the model output, hazard ratio (HR) corresponds to *exp (β)*. HR values > 1 indicate an increased likelihood of an individual leaving a given jurisdiction relative to the reference level (Sweden), while HR < 1 indicate a reduced likelihood. Sweden was set as the reference level because it exhibited the highest hazard ratio, allowing comparisons against the jurisdiction where seals were most likely to leave. Model coefficients were considered statistically significant at *p* < 0.05.

To compare the probability of cross‐boundary haul‐out events over time and across jurisdictions, we generated Kaplan–Meier survival curves using the ‘*survival*’ package (Therneau 2024). Because current software does not support the application of survival functions to mixed effects CPH models, survival curves were based on the simplified fixed effects model. In this context, survival probability at a given time point (in days) represents the likelihood that seals recorded within a particular jurisdiction had not yet undertaken a cross‐boundary haul‐out event.

### 2.4 Spatial Connectivity Analysis

To better understand spatial associations and networks of connectivity across the region, we performed a proximity‐based social network analysis. To achieve this, haul‐out events (i.e., locations) across all individuals were grouped at a 10 km spatial threshold (hereafter referred to as ‘nodes’) using the ‘*spatsoc*’ package (Robitaille et al. 2019). This spatial threshold was considered reasonable given the localised spatial distribution of harbour seals previously recorded in the region (Dietz et al. 2013), and was further validated following visual inspection of haul‐out data presented in this study (refer to Figure 1). To quantitatively examine the spatial network, we calculated several node‐ and edge‐level metrics, which are commonly used for assessing spatial associations and connectivity of ecological networks (Kaur et al. 2024; Nightingale et al. 2023). The edge‐level metrics included: (1) edge density—representing the total number of completed trips that occurred between two haul‐out nodes in the network; (2) edge occupancy—the total number of unique individuals making this connection; and (3) edge weight—a standardisation approach to account for uneven sampling, by taking the average number of trips per individual for each edge (i.e., dividing edge density by edge occupancy). We also calculated two node‐level metrics. The first was node degree—defined as the number of connections an individual haul‐out node had with other nodes in the network. The second node‐level metric was betweenness centrality, calculated using the ‘*igraph’* package (Csárdi et al. 2025). This was based on distance‐corrected edge weights (edge weight divided by the geographic distance between nodes) and normalised to range between 0 and 1, where higher values indicate central nodes in the spatial network that seals must pass to move between other nodes (Wu et al. 2022).

## 3 Results

### 3.1 General Haul‐Out Distribution and Characteristics

Harbour seals in this study exhibited a broad distribution across the Skagerrak–Kattegat region, with haul‐out activity extending from Vest‐Agder in Norway to coastal sites in Denmark (Figure 1). Data from the phone tags identified a total of 1931 haul‐out events across all 26 seals within the Skagerrak–Kattegat region (Figure 1). Deployment duration varied across individuals from 10 to 178 days, and the number of haul‐out events per individual ranged from 2 to 125 (Table 1). Haul‐outs typically occurred within 25 km (mean distance: 16.2 km; 95% CI: 6.7–25.7 km) from the original tagging location, but for some individuals, haul‐outs occurred in more distant locations (Table 1; Figure 1; Figure S1).

**TABLE 1:** Overall‐ and management‐relevant haul‐out characteristics of harbour seals examined in this study (*n* = 26).

Haul‐out characteristics (*n* = 26)	Range	Mean (95% CI)
Overall		
Deployment duration (days)	10–178	103.6 (88.5–118.7)
Number of haul‐outs (#)	2–125	74.3 (58.5–90.1)
Distance from tagging location to most distant haul‐out site (km)	0–260	16.2 (6.7–25.7)
Haul‐out duration (hours)	0.2–27.7	5.8 (5.5–6.0)
Management‐relevant		
Number of management units visited (#)	1–3	
Percentage of haul outs in different management units to where initially tagged (%)	0–90	

*Note:* Range and mean (95% CI) values are calculated across all individuals. The percentage of haul outs in different management unit values is calculated by dividing the number of cross‐boundary haul‐outs by the total number of haul‐out events. Refer to Figure 2 for a visual description of this.

**FIGURE 2:**
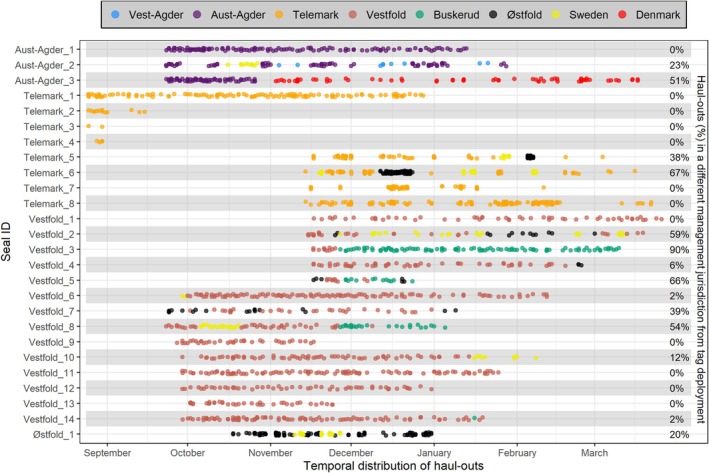
Temporal distribution of haul‐out events for each individual harbour seal (*n* = 26). Harbour seal individuals are labelled (y‐axis) according to the management region where they were initially tagged. Circles represent an individual haul‐out event, and these are coloured by the management unit in which it occurred. Values on the secondary y‐axis display the total proportion (%) of haul‐out events that occurred in a different management unit from where individuals were initially tagged. Vestfold_6 and Vestfold_7 both recorded their first haul‐out in Sweden and Østfold, respectively. Haul outs in Sweden and Denmark occurred in the Swedish Skagerrak and Danish Kattegat management units, respectively (illustrated in Olsen et al. 2014).

Over half of all individuals (54%; *n* = 14) performed haul‐outs in different management areas from where they were initially tagged. Among these individuals, haul‐out events were recorded in up to three distinct management units, although the extent of cross‐boundary activity varied markedly between individuals (Figure 2). Of the 14 seals tagged in Vestfold, nine performed cross‐boundary haul‐outs in Østfold, Buskerud, and/or Sweden. Five of these seals performed repeated (i.e., back‐and‐forth haul‐outs) across these management areas (Figure 2). In contrast, most individuals from Telemark (6 out of 8) restricted their haul‐out activity to within the Telemark management unit. Two Telemark individuals performed haul‐outs in Østfold and Sweden, accounting for 38% and 67% of their haul‐out activity, respectively (Figure 2). Two of the three individuals tagged in Aust‐Agder recorded cross‐boundary haul‐outs in relatively distant locations (100–260 km). One of these two individuals repeatedly switched between haul‐out sites in Vest‐Agder, Aust‐Agder and Sweden, while the other individual switched once, from initially hauling out within Aust‐Agder to subsequently displaying 51% of its haul‐out activity at multiple locations in Denmark (Figures 1 and 2). Of the harbour seals that recorded data during the commencement of the hunting period in Norway (~1 January) (*n* = 21), 7 individuals (33%) hauled out in another management area from where they were initially tagged (Figure S2).

### 3.2 Modelling Results

**FIGURE 3:**
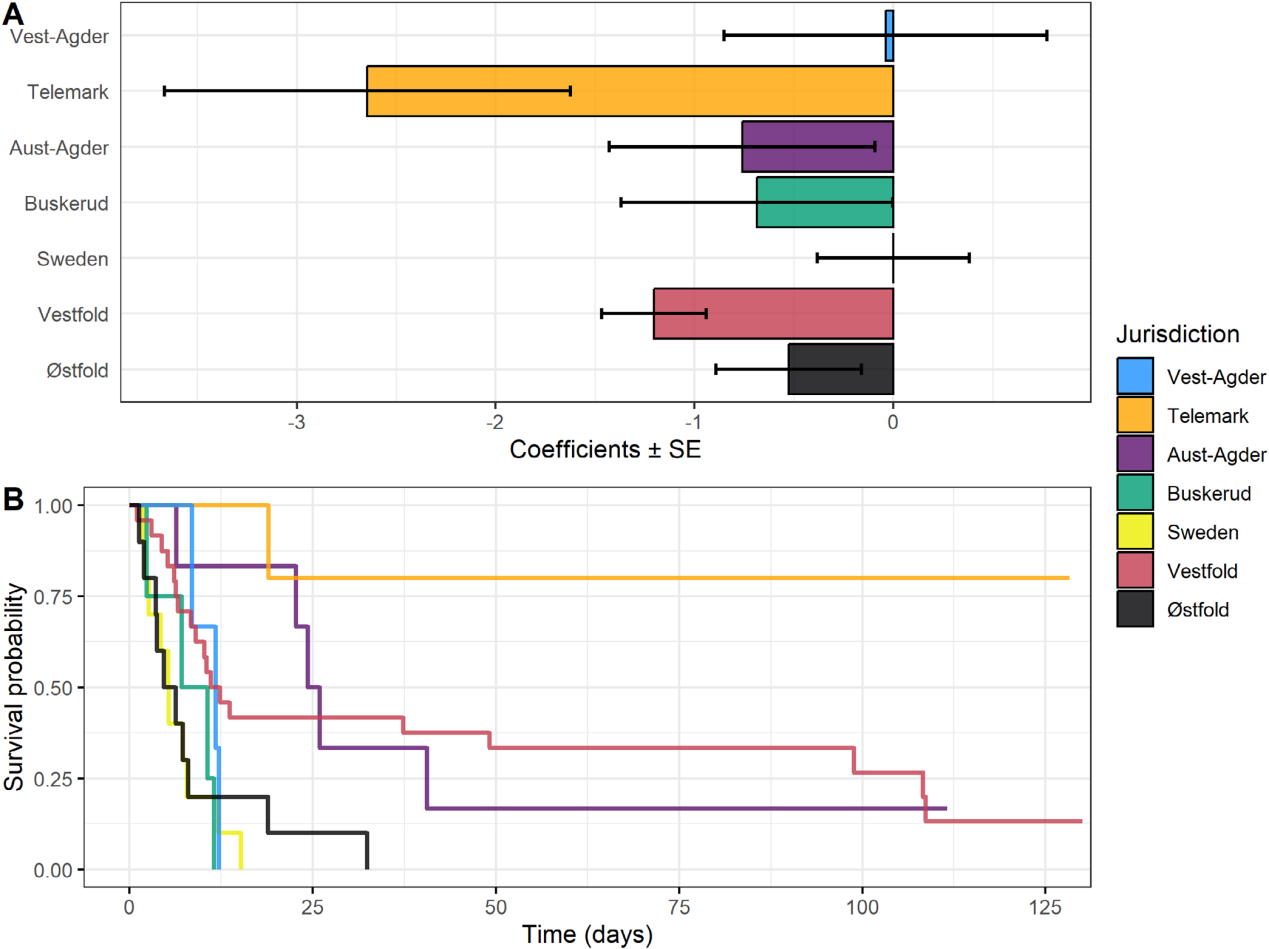
Panel (A) displays the *β* coefficients ± standard error from our Cox proportional hazards mixed effects model showing the likelihood of cross‐boundary haul‐out activity across management jurisdictions. Panel (B) shows the Kaplan–Meier survival curves, illustrating the probability of seals remaining within the same management jurisdiction over time. Steeper declines in survival probabilities reflect a shorter amount of time predicted for seals to perform a cross‐boundary haul‐out.

Our Cox proportional hazards (CPH) model showed jurisdiction‐specific differences in the likelihood of seals performing a cross‐boundary haul‐out (Table 2; Figure 3A). Sweden, as the model reference level, was the jurisdiction where seals had the highest likelihood of performing cross‐boundary haul‐outs. Vest‐Agder, Aust‐Agder, Buskerud and Østfold had hazard ratio (HR) values that were not statistically different from Sweden (*p* > 0.05). However, in Telemark and Vestfold, seals had a significantly lower probability of changing management unit (Table 2; Figure 3A) (*p* < 0.05), suggesting seals in these jurisdictions tended to perform fewer cross‐boundary haul‐outs compared to seals that (temporarily) hauled out in Sweden. Body weight was not a significant predictor of cross‐boundary haul‐out activity (Table 2). The variance associated with seal ID was relatively large, indicating marked variability between individuals (Table 2).

**TABLE 2:** Results of the Cox proportional hazards mixed effects model used to test how the probability of performing a cross‐boundary haul‐out was affected by the departure management jurisdiction and seal body weight.

Predictor variables	Coefficients				
	*β* coefficient	Exp (*β*)	SE (*β*)	*z*‐value	*p*
Jurisdiction: Aust‐Agder	−0.76	0.47	0.79	−0.96	0.34
Jurisdiction: Buskerud	−0.69	0.50	0.80	−0.86	0.39
Jurisdiction: Telemark	−2.65	0.07	1.21	−2.18	**0.03**
Jurisdiction: Vest‐Agder	−0.04	0.96	0.89	−0.04	0.96
Jurisdiction: Vestfold	−1.20	0.30	0.50	−2.42	**0.02**
Jurisdiction: Østfold	−0.53	0.59	0.51	−1.03	0.30
Weight	−0.03	0.97	0.02	−1.57	0.12

Random effects	SD	Variance			
Seal ID	0.99	0.97			

*Note:* The β coefficient represents the log hazard ratio (HR) and exp (*β*) the HR. A *β* coefficient > 0 (HR > 1) indicates an increased likelihood of performing a cross‐boundary haul‐out, while a *β* < 0 (HR < 1) is interpreted as the opposite. These values correspond to the likelihood of performing a cross‐boundary haul‐out compared to the reference category (i.e., Sweden). Larger negative values indicate a higher likelihood of remaining in the initial jurisdiction. Significant *p*‐values (< 0.05) are displayed in bold text.

Kaplan–Meier survival curves further illustrated the jurisdiction and time‐specific differences in cross‐boundary haul‐out activity of seals (Figure 3B). Seals in Telemark had the highest probability of remaining in their original jurisdiction, with survival probabilities exceeding 75% by day 25 and remaining above 75% beyond 100 days. In contrast, seals in all other jurisdictions showed more pronounced declines in survival probability, with median survival times (the time at which 50% of individuals had moved to a different management region) occurring within the first 15–25 days. By day 50, survival probabilities in these jurisdictions had dropped to between 0% and 30%, indicating a high likelihood of cross‐boundary haul‐out activity for seals in these management areas. Overall, the tagging counties (Telemark, Vestfold and Aust‐Agder) exhibited higher site fidelity than the other counties visited during foraging trips, as indicated by the survival curves. Vest‐Agder, Buskerud and Sweden showed particularly low survival probabilities, reflecting their high level of cross‐boundary movements.

### 3.3 Spatial Network Analysis

**FIGURE 4:**
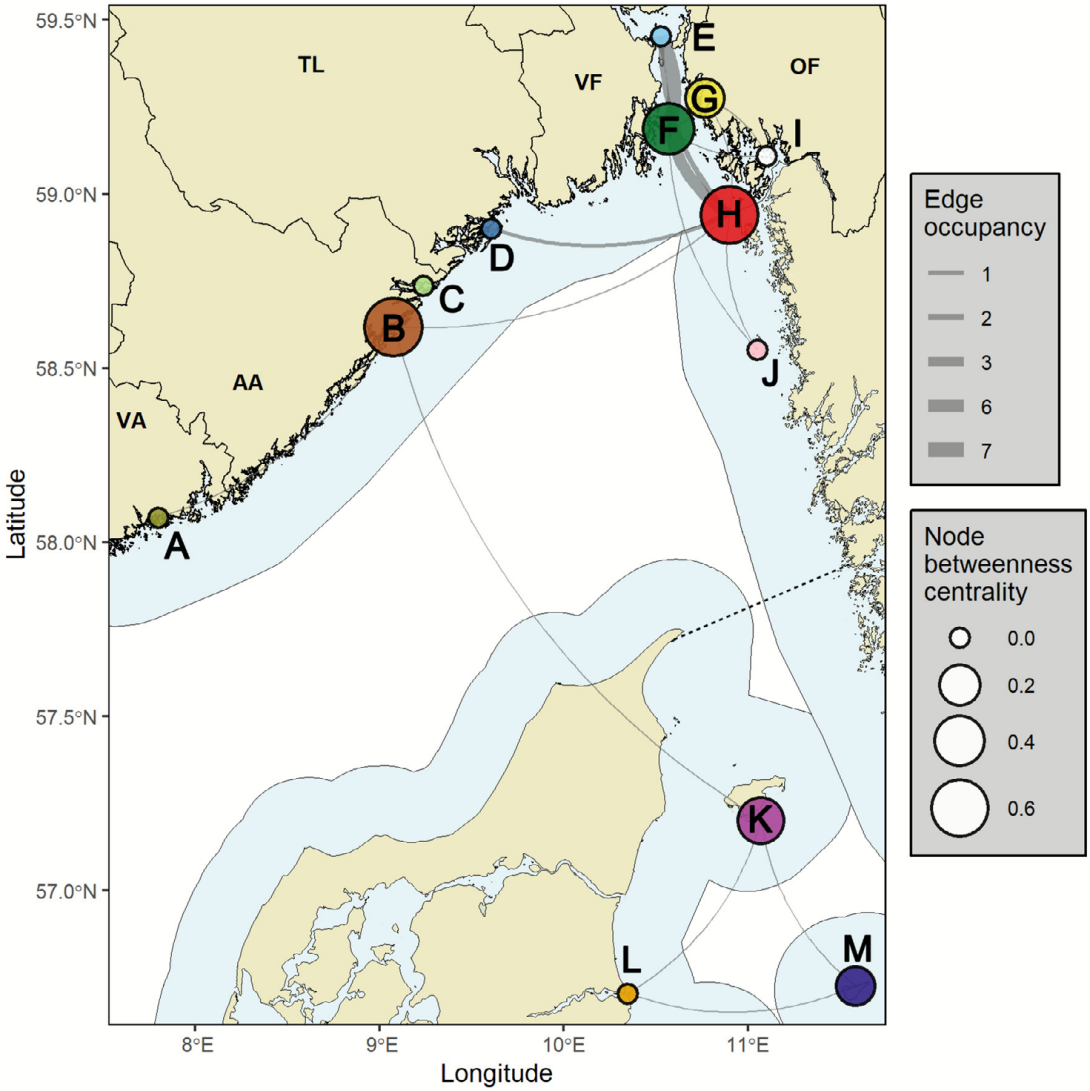
Spatial haul‐out network of harbour seals (*n* = 26) tagged in the Norwegian Skagerrak. Coloured circles represent a distinct haul‐out node identified, based on a 10 km proximity‐based grouping of individual haul‐out locations. Corresponding letters inside the plot represent a unique factor label (A–M) given to each of the 13 different haul‐out nodes. Each node is sized by its calculated betweenness centrality, and the thickness of connecting lines (edges) represents edge occupancy (i.e., number of individuals making this connection). Refer to Section 2.4 for a description of these metrics and Table 3 for a full summary of all edge‐ and node‐level metrics. Mapping features are displayed as in Figure 1.

Our spatial network analysis identified 13 distinct haul‐out nodes distributed across the Skagerrak–Kattegat region, connected by 17 movement corridors (edges) (Table 3; Figure 4). The highest concentration of nodes (*n* = 6) occurred along the stretch of coastline between Buskerud and the northern part of the Swedish west coast (nodes E–J). This region was highly interconnected, with haul‐out nodes connected to 2–6 other nodes in the network (Table 3; Figure 4).

**TABLE 3:** Network edge and node information across the spatial haul‐out network, ordered by edge weight and betweenness centrality scores, respectively.

Network edge information			
Nodes and separation distance (km)	Edge occupancy	Edge density	Edge weight
A ←→ B (96)	1	6	6
F ←→ J (76)	1	6	6
F ←→ G (15)	1	5	5
L ←→ M (76)	1	5	5
F ←→ E (30)	7	34	4.9
K ←→ M (61)	1	4	4
D ←→ H (75)	2	7	3.5
B ←→ C (16)	1	2	2
B ←→ H (111)	1	2	2
H ←→ J (44)	1	2	2
F ←→ H (33)	6	11	1.8
E ←→ H (61)	3	5	1.7
B ←→ K (197)	1	1	1
F ←→ I (32)	1	1	1
H ←→ G (38)	1	1	1
I ←→ G (26)	1	1	1
L ←→ K (71)	1	1	1

Network node information			
Node	No. of individuals	Node degree	Betweenness centrality
B	3	4	0.64
H	11	6	0.62
F	14	5	0.44
K	1	3	0.30
M	1	2	0.17
G	1	3	0.17
D	8	1	0
J	1	2	0
E	7	2	0
C	1	1	0
A	1	1	0
L	1	2	0
I	1	2	0

*Note:* Refer to Section 2.4 for a description of network metrics.

The strongest connections in the network were recorded between node F (the Vestfold tagging site at Bolærne) and nodes E (Buskerud) and H (Hvaler/Koster) (Figure 4). The connection F–E between Vestfold and Buskerud comprised 34 trips across 7 individuals (edge weight = 4.9 trips per individual), reflecting a repeatability in movements within individuals and across multiple individuals. A total of 6 different individuals connected Vestfold node F and node H (encompassing haul‐out sites in Østfold, Norway and the neighbouring Koster Islands in Sweden), though this connection had a more modest edge weight (averaging 1.8 trips per individual, 11 trips in total) (Table 3; Figure 4). Notably, this transnational node H on the border between Norway and Sweden was one of the most influential nodes in the network (betweenness = 0.62), acting as a central hub for 11 individuals, and linking with 6 different nodes in the northern Skagerrak cluster, Sweden (node J) and Telemark (node D—the initial tagging site in Telemark).

Four distinct haul‐out nodes were distributed in the western Skagerrak, between Vest‐Agder and Telemark (nodes A–D) (Figure 4). The tagging site in Telemark (node D) showed a relatively strong connection with node H on the border between Norway and Sweden, with two individuals moving between these nodes. However, both Telemark (node D) and Vest‐Agder (node A) showed betweenness values of 0 as they were only linked with one other node in the network (Table 3). The connection between node A and node B (the initial tagging site in Aust‐Agder) was relatively pronounced (edge weight = 6.0), attributed to strong site fidelity by a single individual. The Aust‐Agder node B was ranked a critical hub in the network (betweenness = 0.64), reflecting its spatial role in linking with other nodes distributed further west and east (including the northern Skagerrak and Danish clusters) (Table 3; Figure 4).

The final three nodes were situated in Danish waters between Læsø (node K), Jylland (node L) and Anholt (node M), resulting from the haul‐out activity of a single individual tagged in Aust‐Agder (node B) (Table 3; Figure 4). This individual transited past nodes H and J as it migrated into Danish waters. All three Danish nodes were connected with each other in a local cluster, which was particularly pronounced between K–M and L–M (edge weight 4.0–5.0).

## 4 Discussion

This study represents one of the first investigations of harbour seal haul‐out site connectivity in southern Scandinavia and is the first to specifically assess movement across administrative management boundaries. We show that harbour seals frequently haul out in different management units, with a high degree of spatial connectivity occurring at regional scales. Our study provides crucial data to improve understanding of harbour seal movement ecology in this data‐limited region, whilst also addressing an important applied issue regarding the efficacy of administrative management boundaries in the Norwegian Skagerrak. More broadly, this work demonstrates how telemetry‐based assessments of spatial connectivity can be used to evaluate and refine management frameworks for other wide‐ranging marine species that routinely cross jurisdictional boundaries.

### 4.1 Spatial Distribution and Connectivity

Information on seasonal distribution and haul‐out activity of harbour seals is critical for better understanding population structure and connectivity, and to describe some of the demographic processes that contribute to shaping population dynamics (Carroll et al. 2020; Carter et al. 2022). In this study, harbour seal haul‐out activity was broadly distributed across the Skagerrak–Kattegat region. Although most individuals predominantly used relatively local‐scale haul‐out sites (< 50 km from their initial tagging location), several individuals displayed longer‐range and more extensive movements between haul‐out sites (> 100 km). These findings align with numerous other studies conducted in European waters, which describe harbour seals as a generally localised species, but capable of regional‐scale dispersal (Carroll et al. 2020; Dietz et al. 2013; Vincent et al. 2017; Sharples et al. 2012; Jones et al. 2015).

Satellite telemetry studies examining the connectivity of harbour seals between haul‐out sites in European waters have yielded variable results. In France, individuals exhibit little to no movement between haul‐out sites, suggesting discrete and highly localised populations (Vincent et al. 2017). However, in the United Kingdom, extensive tracking efforts have shown regional‐scale movements and spatial connectivity between haul‐out sites (Carter et al. 2022; Sharples et al. 2012). Our findings demonstrate that harbour seals in the Skagerrak–Kattegat region similarly display high levels of regional connectivity, with individuals using numerous haul‐out sites across an integrated spatial network.

The northeast Skagerrak, consisting of the Oslofjord (Vestfold, Buskerud and Østfold) and the Swedish coastal areas, contained a dense and highly connected cluster of haul‐out sites. This area formed a central hub of connectivity across the region, with individuals frequently transitioning between multiple haul‐out nodes and administrative management units. This supports ongoing genetic analyses of harbour seals in Norway, which indicate that the Oslofjord area comprises a single genetic unit (M. Quintela, personal communication). Individuals tagged in Aust‐Agder and Telemark also hauled out at sites within this northeast Skagerrak cluster, highlighting its potential role as a focal area of population connectivity. These spatial dynamics may reflect the presence of favourable foraging grounds. Following major declines in large predatory fish populations, the area now appears to support a rich community of small fish (Synnes et al. 2023), which are common prey for harbour seals (Sørlie et al. 2020). Additionally, seal movements in the area may be influenced by social and demographic processes associated with population growth and recovery after historical exploitation and disease outbreaks, followed more recently by declines linked to compound environmental stressors (Carroll et al. 2025).

In this study, one individual tagged in Aust‐Agder made extensive movements to haul‐out sites in Denmark, located over 260 km away. These results provide the first empirical evidence of harbour seal haul‐out connectivity between sites in the Norwegian Skagerrak and Danish Kattegat. This individual was a large male, potentially suggesting it is an animal that breeds in Danish waters but frequents Norwegian waters during feeding excursions. If this is a common movement strategy, these findings have important implications for our understanding of harbour seal movement ecology in the region, particularly in the context of wildlife epizootics and pathogen spread. The Danish island of Anholt—a haul‐out node in this study—has previously been identified as the epicentre of several major disease outbreaks which subsequently spread throughout southern Scandinavian and European waters (Hall et al. 2006; Dietz et al. 2013). While previous harbour seal telemetry studies in the region have described at‐sea movement pathways stemming from Anholt (Dietz et al. 2013), connectivity between coastal haul‐out sites has not specifically been examined. This is important because among central‐place and colonial breeding pinnipeds, haul‐out behaviours are believed to play a significant role in disease transmission and pathogen spread (Riaz et al. 2024). By identifying networks of spatial connectivity between haul‐out sites, our findings provide critical information needed to inform population monitoring and biological risk assessment in the context of pathogen spread.

Contrary to these connectivity patterns displayed across the Skagerrak–Kattegat network, individuals tagged in Telemark were relatively sedentary throughout the tracking period and had the highest site (county) fidelity. Their relatively short track durations (refer to Table S1) may partly contribute to this pattern. Vestfold was the county with the second largest site fidelity, which could be tied to locally favourable foraging and/or social conditions. Not surprisingly, all tagging counties (Telemark, Vestfold and Aust‐Agder) showed larger site fidelity compared to other counties visited during foraging trips, as shown by the survival curves. In any case, our regionally variable results highlight how harbour seal behaviours, connectivity and habitat preferences can vary markedly over small spatial scales, with differences likely influenced by both intrinsic (e.g., age class, sex, individual experience) and extrinsic factors (e.g., prey availability, social dynamics, anthropogenic activity) (Carter et al. 2022; Russell et al. 2015; Vincent et al. 2017).

### 4.2 Movement in the Context of Norwegian Management Boundaries

The delineation of harbour seal management units along county‐level administrative boundaries in Norway has offered a pragmatic framework for 15 years, particularly given the paucity of spatially resolved seal population and movement data. However, our findings suggest that these boundaries do not always align with the actual movement and dispersal patterns of harbour seals in the Skagerrak, where counties are more numerous and relatively small compared to those on the Norwegian west coast and in northern Norway. Satellite tracking of harbour seals in Nordland and Porsangerfjord (two counties in northern Norway) showed that individuals had a high site fidelity, largely remaining near their tagging sites (Ramasco 2008; Ramasco et al. 2017). In contrast, we found that harbour seals in the Norwegian Skagerrak had a strong tendency to perform cross‐boundary haul‐out events, frequently and repeatedly transitioning between different management jurisdictions. In fact, most individuals were predicted to haul out in different management units within the first 25 days of tag deployment. Consequently, by the time Norway's annual hunting season begins in January, many harbour seals may be hauled out in jurisdictions other than where they were originally counted several months earlier during the moulting period (~ August). This potential misalignment between harbour seal management boundaries and haul‐out activity in the Skagerrak raises concerns for the efficacy of Norway's county‐based harvesting quotas and population estimates. The current annual hunting quota for harbour seal across the Norwegian Skagerrak is 17 individuals in Telemark, 50 in Vestfold, 52 in Østfold and none in Aust‐Agder and Vest‐Agder given the current population numbers (LOVDATA 2025). However, our study demonstrates that under the current management regime, individuals hunted in one management unit may have originated from another, undermining county‐level regulatory efforts.

Evidently, there is a need to re‐evaluate harbour seal management boundaries and hunting practices in Norway to better reflect biologically meaningful population structuring. Similar to the Norwegian management framework, harbour seal populations in the United Kingdom are defined and regulated at a sub‐national scale. However, a key distinction is that management units are informed by ecological data derived from extensive aerial surveys, satellite telemetry programs and population genetic studies (Olsen et al. 2017; Sharples et al. 2012; Thompson et al. 2019). This approach has facilitated a more robust and adaptive management approach to spatially explicit threats and demographic trends (Blanchet et al. 2021). A similar ecologically defined framework, supported by information from ongoing genetic studies (IMR, unpublished) and additional satellite tracking data, could be pursued in Norway to better align management strategies with the spatial ecology of harbour seals.

Beyond Norwegian regulation, there is also a need for transnational coordination in the management of harbour seals across southern Scandinavia. It is important to highlight that one of the haul‐out areas (node H) identified in this study spans both Norwegian and Swedish waters, encompassing closely situated haul‐out sites in Hvaler (Norway) and Koster Islands (Sweden). On the Norwegian side, there is a hunting quota in place that allows for the removal of harbour seals (LOVDATA 2025). But just across the border on the Swedish side, harbour seals are protected within the Kosterhavet National Park. Our findings suggest that seals originating from Sweden may perform foraging trips across different Norwegian Skagerrak jurisdictions, where they could be subject to Norwegian hunting quotas. This regulatory disparity underscores the need for more cohesive and collaborative management of harbour seal populations in the region, as a shared natural resource.

### 4.3 Future Research Direction

It is important to acknowledge that the results of this study are based on a relatively small harbour seal telemetry dataset, compared to studies in other European regions (Carter et al. 2022; Jones et al. 2015). However, even with this modest sample size of 26 individuals tagged over a 6‐year period from 2017 to 2022, we were able to identify substantial individual variability in haul out behaviours. Over half (54%) of all seals performed repeated, and at times, extensive cross‐boundary movements, while the other half remained within a single management unit throughout the tracking period, and this pattern was consistent within tagging sites. Considerable individual‐specific heterogeneity is commonly reported in harbour seal telemetry studies (Russell et al. 2015; Steingass et al. 2019) and is a valuable ecological finding in itself, reflecting the various intrinsic and extrinsic factors that shape movement decisions (Hertel et al. 2020; Shaw 2020).

This variability, however, presents analytical challenges, particularly when drawing population‐level inferences. In this study, we incorporated seal ID as a random effect within our model structure—a standard approach to account for individual‐level variation in movement behaviour (Hertel et al. 2020; Muff et al. 2020). While this improves our capacity to make population‐level inferences with the available dataset, it does not change the fact that the sample size is relatively small, and that the efforts partly targeted seals in different jurisdictions in different years. This highlights the importance of conducting larger‐scale, repeated telemetry studies with more balanced sampling across sex and weight classes, and also covering the spring and summer seasons. Such studies would enable robust testing of intrinsic influences on movement behaviour and connectivity, whilst also disentangling these effects from spatial, seasonal and annual variability in population‐level movement patterns.

Critically, future research should also prioritise collecting data from Swedish harbour seals. The harbour seal abundance in the Swedish Skagerrak is markedly higher than along the Norwegian Skagerrak coast (Carroll et al. 2025; ICES 2023), and individuals from these colonies may temporarily use Norwegian waters. Integrating data from harbour seals in Sweden would improve our capacity to understand transnational movement dynamics, thereby supporting the development of coordinated management strategies across national borders.

Despite these limitations, this study contributes critical insights to improve our understanding of harbour seal haul‐out activity in southern Scandinavia and provides important management‐relevant information. Future research efforts should prioritise collecting complementary telemetry data across the region. Importantly, these data can also be integrated with ongoing genetic works, providing complementary information on inter‐generational population structure and intra‐generational demographic connectivity needed to inform the development of robust and ecologically relevant management frameworks (Müller et al. 2023).

## 5 Conclusion

This study provides the first empirical evidence of haul‐out site connectivity among harbour seals in southern Scandinavia, including movements across administrative boundaries in the Norwegian Skagerrak, as well as beyond national borders. By showing that individuals routinely transition between multiple national and sub‐national management units, our findings demonstrate that current administrative boundaries do not adequately reflect the spatial ecology of harbour seals. More broadly, these results underscore the need to integrate telemetry‐based insights into area‐based management frameworks, particularly in regions where highly mobile species intersect with small‐scale jurisdictions.

## Author Contributions

**Javed Riaz:** conceptualization (equal), data curation (lead), formal analysis (lead), methodology (equal), visualization (lead), writing – original draft (lead), writing – review and editing (lead). **Kjell T. Nilssen:** funding acquisition (equal), investigation (equal), writing – review and editing (supporting). **Martin Biuw:** investigation (equal), writing – review and editing (supporting). **Even Moland:** funding acquisition (equal), investigation (supporting), writing – review and editing (supporting). **Michael Poltermann:** investigation (equal), data curation (supporting). **Martin Kristiansen:** investigation (equal). **Carla Freitas:** conceptualization (equal), funding acquisition (supporting), investigation (equal), data curation (supporting), formal analysis (supporting), methodology (equal), project administration (lead), writing – review and editing (supporting).

## Conflicts of Interest

The authors declare no conflicts of interest.

## Supporting information


**Appendix S1:** ece372718‐sup‐0001‐AppendixS1.docx.

## Data Availability

All data and R code supporting this study are publicly available on GitHub: https://github.com/Javedmoves/.
