# Supplementary material for: Haul‐Out Site Use and Connectivity of Harbour Seals Between Management Units in Southern Scandinavia
Source: Ecol Evol. 2026 Jan 13;16(1):e72718. doi: 10.1002/ece3.72718 (PMC12796850; doi:10.1002/ece3.72718)
Supplement: Supplementary file 1 — Appendix S1: ece372718‐sup‐0001‐AppendixS1.docx. [file ECE3-16-e72718-s001.docx]

**SUPPLEMENTARY MATERIAL**

Table S1 – Deployment details for each of the 28 harbour seals tagged with GPS phone tags between 2017 and 2022. Details include unique seal ID (based on deployment management location), weight, sex, tagging date and tag duration. The * symbol indicates the two individuals that were excluded from our haul-out and spatial connectivity analyses due to short tag duration.

| **Original seal**  **ID** | **Revised seal ID** | **Tagging**  **site** | **Weight (kg)** | **Sex** | **Tagging date** | **Tag duration (days)** |  |
| --- | --- | --- | --- | --- | --- | --- | --- |
|  |  |  |  |  |  |  |  |
| pv76-M78_Rulle-20 | Aust-Agder_1 | Askerøy | 75 | M | 23/09/2021 | 114 |  |
| pv76-M83_Ra-20 | Aust-Agder_2 | Askerøy | 80 | M | 23/09/2021 | 127 |  |
| pv76-M94_Morene-20 | Aust-Agder_3 | Askerøy | 91 | M | 23/09/2021 | 178 |  |
| pv35b-05-11 | Telemark_1 | Jomfruland | 59 | F | 25/08/2017 | 128 |  |
| pv35b-06-11 | Telemark_2 | Jomfruland | 72 | M | 25/08/2017 | 22 |  |
| pv35b-08-11 | Telemark_3 | Jomfruland | 72 | M | 25/08/2017 | 10 |  |
| pv35b-10-11 | Telemark_4 | Jomfruland | 31 | F | 28/08/2017 | 63 |  |
| pv74-F46_Olivia-20 | Telemark_5 | Jomfruland | 43 | F | 14/11/2020 | 122 |  |
| pv74-M70_Osito-20 | Telemark_6 | Jomfruland | 67 | M | 14/11/2020 | 126 |  |
| pv74-M86_Bjorn-20 | Telemark_7 | Jomfruland | 83 | M | 14/11/2020 | 90 |  |
| pv74-M88_Diego-20 | Telemark_8 | Jomfruland | 85 | M | 14/11/2020 | 139 |  |
| pv35b-09-11 | Telemark_9 * | Jomfruland | 82 | M | 25/08/2017 | 1 |  |
| pv35b-04-11 | Telemark_10 * | Jomfruland | 87 | M | 25/08/2017 | 3 |  |
| pv68-F53_Iris-14 | Vestfold_1 | Bolærne | 50 | F | 17/11/2019 | 136 |  |
| pv68-F56_Karin-14 | Vestfold_2 | Bolærne | 53 | F | 15/11/2019 | 128 |  |
| pv68-M40_Pedro-14 | Vestfold_3 | Bolærne | 37 | M | 17/11/2019 | 119 |  |
| pv68-M42_Einar-14 | Vestfold_4 | Bolærne | 39 | M | 17/11/2019 | 106 |  |
| pv68-M47_Vemund-14 | Vestfold_5 | Bolærne | 44 | M | 17/11/2019 | 42 |  |
| pv77-023-20 | Vestfold_6 | Bolærne | 67 | M | 29/09/2022 | 138 |  |
| pv77-028-20 | Vestfold_7 | Bolærne | 75 | M | 24/09/2022 | 112 |  |
| pv77-029-20 | Vestfold_8 | Bolærne | 73 | M | 23/09/2022 | 107 |  |
| pv77-030-20 | Vestfold_9 | Bolærne | 86 | M | 27/09/2022 | 58 |  |
| pv77-064-21 | Vestfold_10 | Bolærne | 79 | M | 29/09/2022 | 135 |  |
| pv77-065-21 | Vestfold_11 | Bolærne | 62 | M | 29/09/2022 | 120 |  |
| pv77-066-21 | Vestfold_12 | Bolærne | 81 | M | 29/09/2022 | 95 |  |
| pv77-067-21 | Vestfold_13 | Bolærne | 87 | M | 02/10/2022 | 58 |  |
| pv77-068-21 | Vestfold_14 | Bolærne | 36 | F | 29/09/2022 | 115 |  |
| pv74-M62_Gamle-Erik-20 | Østfold_1 | Hvaler | 59 | M | 20/10/2020 | 106 |  |


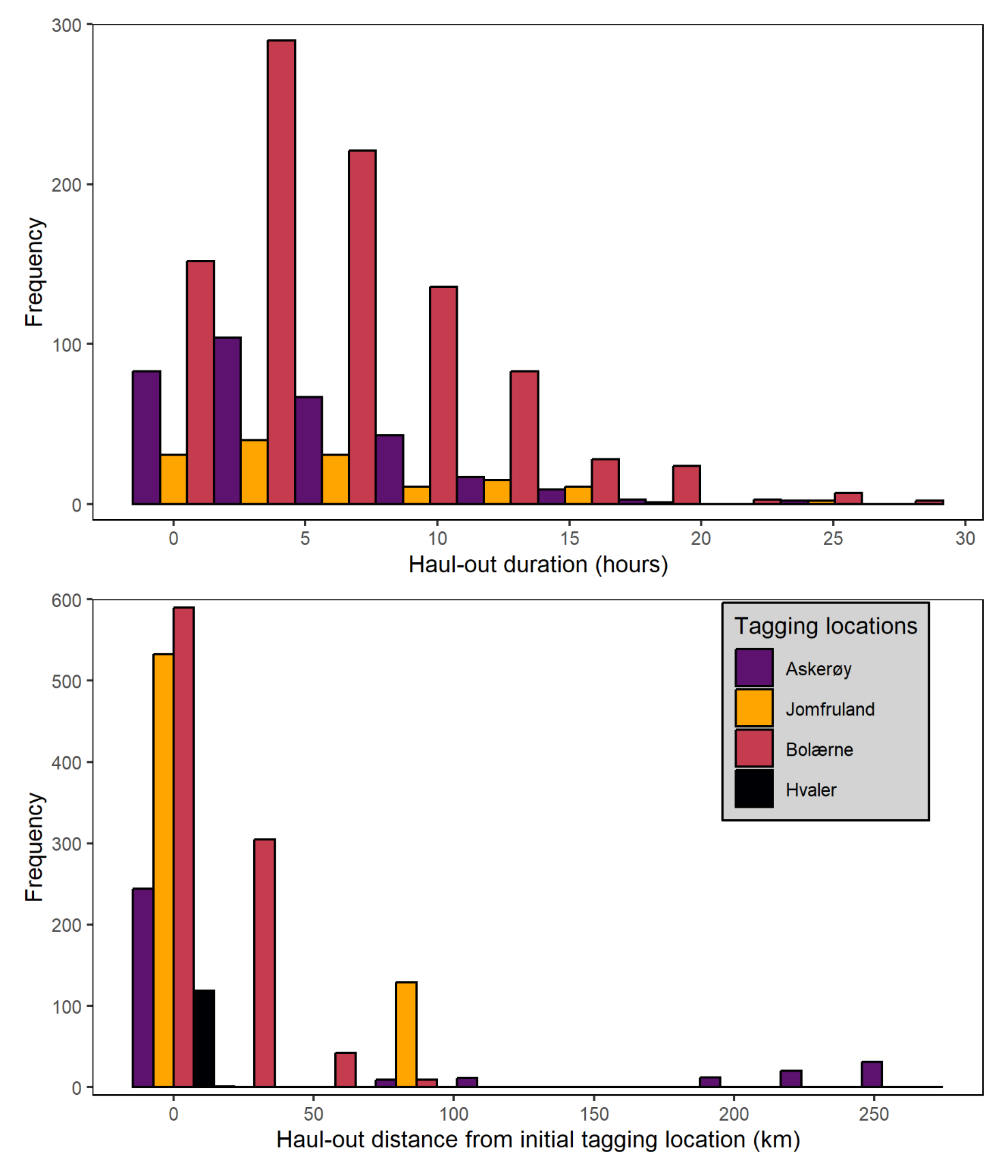


Fig. S1 – The frequency distribution of haul outs in relation to haul-out duration (hours) and distance from initial tagging location (km). Due to technical issues with the GPS phone tags deployed in 2020, haul-out duration data from these individuals has been omitted from summary calculations and this frequency distribution plot. Data displayed are coloured by seal tagging location.


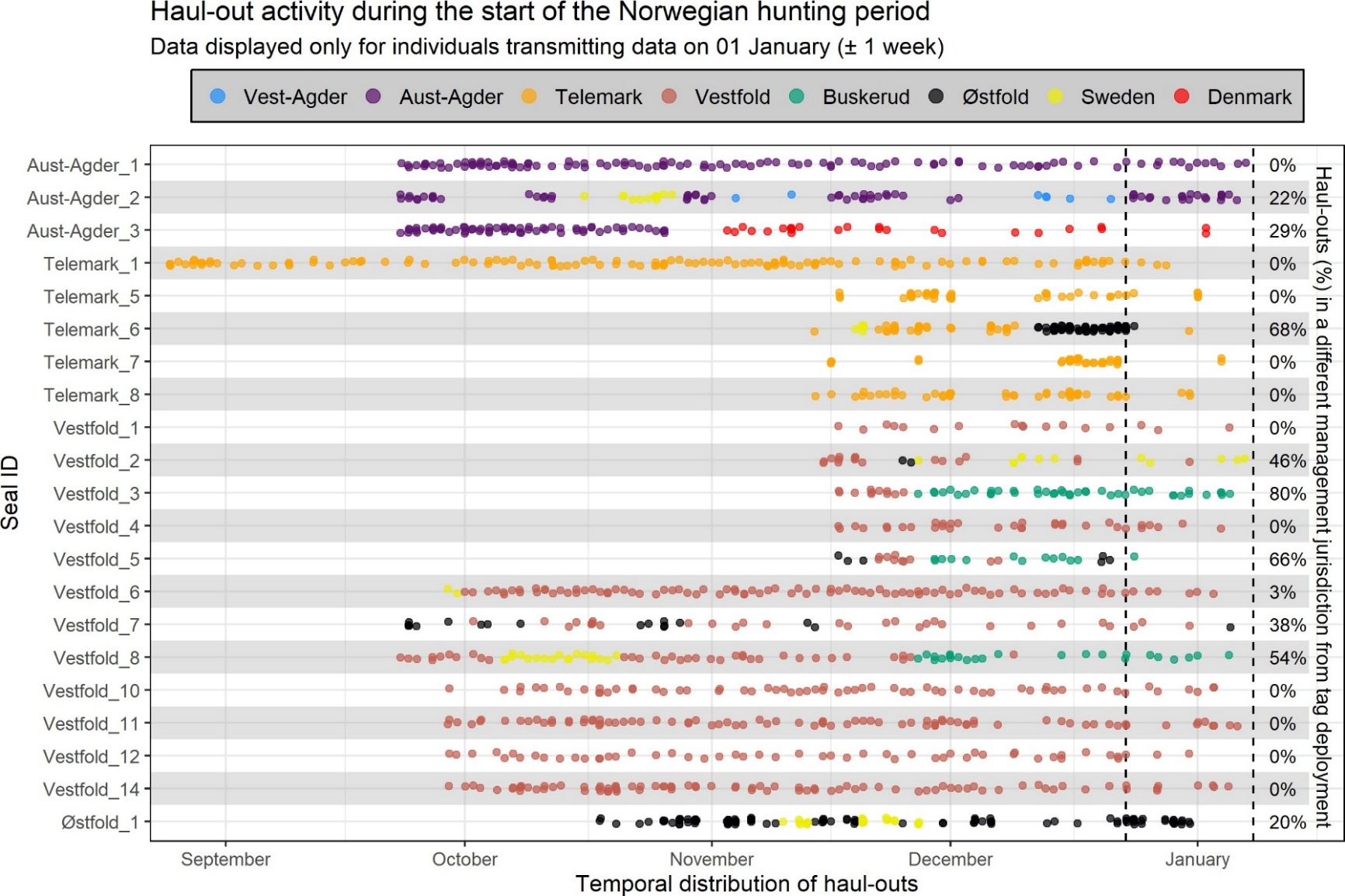


Fig. S2 - Temporal distribution of haul-out events for individual harbour seals which recorded data on 01 January ± 1 week (i.e. coinciding with the start of the harbour seal hunting period in Norway). Data are displayed consistently with Fig. 2; however, proportion values reflect cross-boundary movements only up to 07 January.
